# Hydrocele of the canal of Nuck mimicking an inguinal hernia in a 34-year-old pregnant woman: a case report

**DOI:** 10.11604/pamj.2025.51.36.47817

**Published:** 2025-06-09

**Authors:** Celsus Ukelina Undie, Kenechi Stanislaus Nedosa, Uchechukwu George Ogbu, Adeyemi Oluwatobi Akano, Adeyemi Odunayo Precious

**Affiliations:** 1 Department of Urology, Kelina Hospital, Abuja, Nigeria; 2 Clinical Department, Kelina Hospital, Abuja, Nigeria; 3 Department of Obstetrics and Gynaecology, Kelina Hospital, Abuja, Nigeria; 4 Clinical Research Department, Kelina Hospital, Abuja, Nigeria

**Keywords:** Hydrocele, canal of Nuck, inguinal swelling, misdiagnosis, case report

## Abstract

Hydrocele of the canal of Nuck (HCN) is a rare condition in females that commonly presents in infancy and early childhood. It occurs when fluid accumulates within a patent part of the processus vaginalis causing a swelling in the groin. Clinicians would typically not consider it while evaluating inguinal swellings in adult females. Consequently, patients are likely to remain undiagnosed until complications set in. Herein, we present a clinically rare case of HCN in a pregnant adult female. We also aim to report how easily the diagnosis was missed, and to remind the medical community of other likely causes of inguinal swelling that could mimic an inguinal hernia. We managed a gravid 34-year-old university graduate with a spontaneous swelling on the left inguinal region. The swelling was a painless, firm, irreducible mass of 3 months’ duration. She was gravida 1, para 0, in her first trimester. There was no associated symptom. Abdominal ultrasound scan found a well-defined mass of size 2.30 x 3.88cm around the left Hesselbach's triangle containing homogenous fluid, with minimal vascularity on colour Doppler interrogation. The working diagnosis was irreducible left inguinal hernia in a pregnant woman. This was later discovered to be a misdiagnosis. She was booked for inguinal herniorrhaphy, but HCN was found intraoperatively. Diagnosis of HCN is a challenge as it is seldom made on the basis of clinical findings alone. A high index of suspicion should be entertained by clinicians for early diagnosis and appropriate management.

## Introduction

In 1691, Anton Nuck, a Dutch anatomist, first described the canal of Nuck, which is the processus vaginalis within the inguinal canal of females [1]. It is a small evagination of the parietal peritoneum that is attached to the uterus by the round ligament through the internal inguinal ring into the inguinal canal. This structure is similar to the processus vaginalis in males [2]. When there is partial or complete failure of obliteration of the processus vaginalis, the result is formation of a potential space which is the canal of Nuck [3]. In adult females, hydrocele of the canal of Nuck is a very rare condition that results from failure of obliteration of the distal portion of the canal, which forms a fluid-containing sac [4]. While preoperative diagnosis of this type of hydrocele is important, the diagnosis of hydrocele of the canal of Nuck is seldom made based on clinical findings alone [2] and should be considered when diagnosing inguinal swellings in female patients. Hydrocele of the canal of Nuck is a rare entity, with literature estimating the incidence at 1% in young girls [5]. The incidence in adult females has not been established due to its rarity [6]. In most cases, the canal of Nuck is obliterated before birth. The cause of this obliteration is unknown, but some studies show that calcitonin gene-related peptide (CGRP) that is released from the genitofemoral nerve plays a role [5]. In some cases, there is incomplete obliteration or even no obliteration at all. This can cause a cyst of the canal of Nuck or even a congenital (communicating) hernia inguinalis. The fluid inside the cyst of this canal is a result of imbalance between production and absorption of fluid by the parietal peritoneum [7] which lines the cyst. When an inguinal mass presents with features of inguinal hernia such as reducibility or discomfort especially with coughing, during exercise or bowel movements, diagnosis is easier to make as the suspicion of hernia is higher. However, in the absence of these symptoms, diagnosis would be more difficult. Preoperative diagnosis of this type of hydrocele can prove extremely difficult as it can easily mimic an inguinal hernia [8].

This is a challenge as there are many differentials for masses of the inguinal region, e.g. lipomas, leiomyoma and endometriosis of the round ligament [8]. The most common diagnostic modalities are not very sensitive in distinguishing hydrocele of the canal of Nuck from other cystic swellings in the inguinal canal, as even inguinal hernias with bowel contents sometimes contain fluid. A high index of suspicion is therefore needed for early diagnosis and proper management. An irreducible inguinal hernia is different from a strangulated hernia. Inguinal hernias are at risk of irreducibility or incarceration, which may result in strangulation and obstruction; however, unlike with femoral hernias, strangulation of inguinal hernia is rare [9]. If the contents of the hernia cannot be reduced, the hernia is considered incarcerated. A strangulated hernia occurs when the hernia contents are ischemic due to a compromised blood supply [10]. Irreducible inguinal hernia is not always tender or painful and could also contain fluid [10]. Presence of fluid in both HCN and inguinal hernias, and the rarity of HCN makes it clinically difficult to distinguish between inguinal hernias and HCN. The actual incidence of inguinal hernia in females is not known, but it is more common than hydrocele of the canal of Nuck. For this reason, clinicians are more familiar with inguinal hernias. As a result, many patients remain undiagnosed until complications begin to set in. In the reports reviewed, majority of cases were diagnosed intraoperatively. The incidence of missed diagnosis was a motivating factor for every surgeon who reported hydrocele of the canal of Nuck in the literature, in the hope that other clinicians would not as well miss the diagnosis subsequently. The authors considered it important to report this case to create awareness that hydrocele of the canal of Nuck can be seen even in our environment. If diagnosis had been made in this patient pre-operatively, surgery would have been delayed until after parturition. Because HCN was misdiagnosed as a hernia, surgery was considered urgent, to avoid obstruction or strangulation as the pregnancy progressed.

## Patient and observation

**Patient information:** a 34-year-old gravid woman, university graduate and in her first trimester of pregnancy, presented to our facility with a 3-month history of a spontaneous, painless swelling in the left inguinal region. She reported no associated symptoms such as nausea, vomiting, constipation, or lower urinary tract symptoms. There was no history of prior inguinal surgery or trauma. She was otherwise healthy, with unremarkable vital signs and no comorbidities.

**Clinical findings:** on examination, the swelling appeared firm, irreducible, and non-tender. It was noticeable in the supine position but tended to be less visible or palpable in the standing position. The mass was not compressible or reducible.

**Timeline of current episode:** the patient first noticed a painless swelling in her left inguinal region approximately three months prior to presentation. Over this period, the swelling remained unchanged in size. At the time of presentation, she was in her first trimester of pregnancy. Given the physical findings and suspicion for an irreducible left inguinal hernia, a preoperative ultrasound was performed, which revealed a well-defined cystic mass. Despite imaging, the diagnosis of hydrocele of the canal of Nuck was not considered preoperatively, and the patient was scheduled for surgical exploration. The definitive diagnosis was made on the operating table. The patient did well post-operatively and was discharged 2 days after surgery. She presented again one week after discharge with no fresh complaints.

**Diagnostic assessment:** an abdominal ultrasound revealed a well-defined mass measuring 2.30 x 3.88 cm located around the left Hesselbach´s triangle containing homogenous fluid, with minimal vascularity on colour Doppler interrogation. Despite these findings, the mass was interpreted preoperatively as an irreducible inguinal hernia due to the typical location and the rarity of hydrocele of the canal of Nuck.

**Figure 1:**
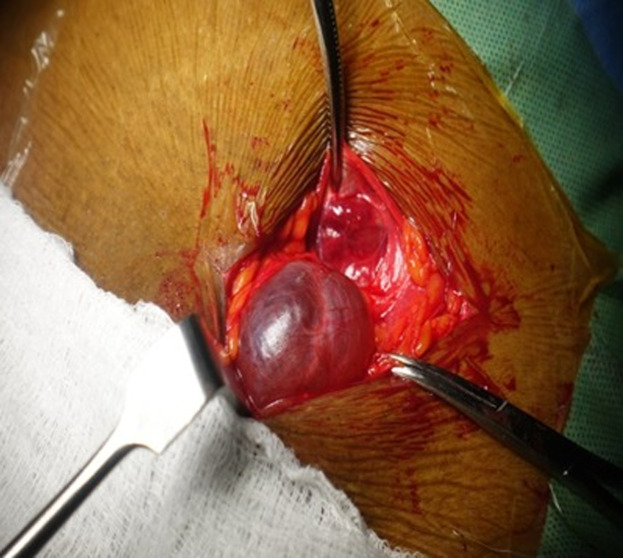
hydrocele of the canal of Nuck, exposed at surgery

**Figure 2:**
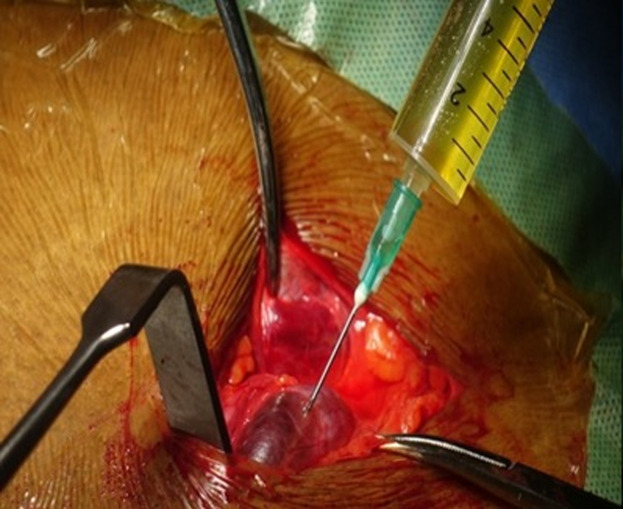
aspirated clear, serous fluid, similar to peritoneal fluid

**Figure 3:**
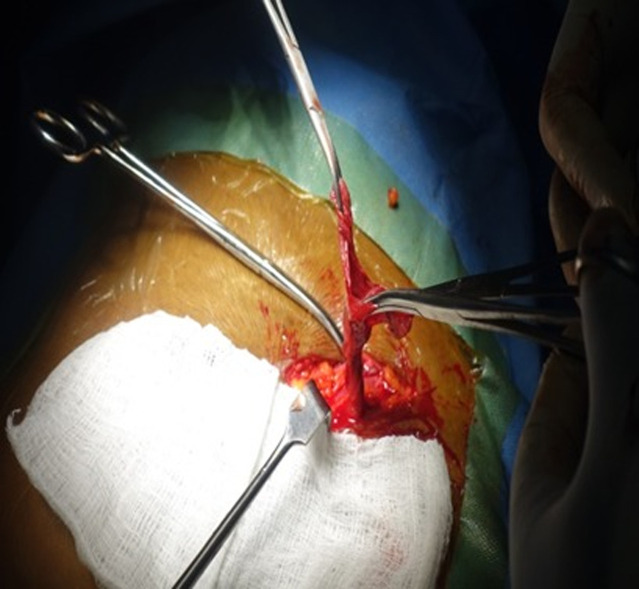
cyst aspirated and sac opened to find cavity which did not have any external communication

**Diagnosis:** the definitive diagnosis of hydrocele of the canal of Nuck was made intraoperatively. During surgical exploration, the sac of the swelling containing about 40 ml of clear serous fluid within the cyst cavity was dissected from the inguinal canal (Figure 1). The cyst was dissected from the round ligament. It was aspirated, and the sac opened to find that the cavity did not have any external communication (Figure 2, Figure 3), which is consistent with hydrocele of the canal of Nuck.

**Therapeutic interventions:** under regional (subarachnoid block) anesthesia, the cyst was dissected from the round ligament. It was aspirated, and the sac opened to find that the cavity did not have any external communication (Figure 2, Figure 3), which is consistent with hydrocele of the canal of Nuck. The cyst was excised and part of the round ligament of the uterus was also excised. The inguinal canal was closed around the round ligament. The wound was closed in layers, with subcutaneous Vicryl to skin. The procedure was well tolerated.

**Follow-up and outcome of interventions:** the patient was monitored postoperatively and discharged after two days. Although this surgery could be a day case, the patient´s hospital stay was extended because of the pregnancy. She returned for follow-up one week later with no new complaints. The surgical wound had healed well without any complications. She was subsequently referred back to her obstetrician for continued antenatal care. No recurrence or postoperative complications were reported at follow-up.

**Patient perspective:** patient was satisfied with her management. She received throughout the process; she returned to her antenatal care without any issues.

**Informed consent:** written informed consent was obtained and documented with date and time from the patient for publication and any accompanying images and they are available upon request.

## Discussion

A crucial step in expanding differential diagnosis associated with groin masses is the proper understanding of the anatomy and development of hydrocele of the canal of Nuck, which can be mistaken for common inguinal hernias, abscesses, or tumours [9]. According to Lucas, different radiological modalities such as computed tomography (CT), magnetic resonance imaging (MRI) and us can be used individually or in combination to provide diagnostic support for hydrocele of the canal of Nuck as opposed to other entities on the differential. Hydrocele of the canal of Nuck will typically show various characteristics on ultrasound scan, including minimal vascularity on colour Doppler interrogation [9], which is consistent with our findings. This report shows that diagnosis of hydrocele of the canal of Nuck could be challenging, as it was missed even in the hands of an experienced surgeon of over 25 years in practice. Its presentation mimicked that of common inguinal swellings as it was a painless, firm, irreducible mass with an associated swelling noticeable in a supine position. The patient had a painless, firm and irreducible swelling on the left inguinal region with no history of constipation, nausea, vomiting or urinary tract symptoms. Initial review suggested an irreducible inguinal swelling in a pregnant woman. We confirmed hydrocele of the canal of Nuck intraoperatively. This feature was consistent with majority of reported cases in the literature [5-7], where hydrocele of the canal of Nuck was not conclusively diagnosed until surgery was performed.

## Conclusion

Diagnosis of hydrocele of the canal of Nuck is a challenge, as it is seldom made based on clinical findings alone. This is a case of a misdiagnosis and this report is an effort to prevent such from happening in similar cases. The unique feature of this case was that the patient was a pregnant woman and the surgeon was concerned about leaving an inguinal hernia to progress beyond the first trimester into the third term, where it could pose a higher risk as the pregnancy progressed, with chances of obstruction increasing with increased intra-abdominal pressure. More efforts, including correlating clinical findings with ultrasound reports, should be made to arrive at appropriate diagnosis and treatment options when inguinal swellings are seen in the surgical clinic. A high index of suspicion should also be maintained by clinicians for early diagnosis and appropriate management.
